# Survival before and after the introduction of pertuzumab and T-DM1 in HER2-positive advanced breast cancer, a study of the SONABRE Registry

**DOI:** 10.1007/s10549-021-06178-8

**Published:** 2021-03-20

**Authors:** Khava I. E. Ibragimova, Sandra M. E. Geurts, Sander Croes, Frans Erdkamp, Joan B. Heijns, Jolien Tol, Birgit E. P. J. Vriens, Kirsten N. A. Aaldering, Marcus W. Dercksen, Manon J. A. E. Pepels, Natascha A. J. B. Peters, Linda van de Winkel, Dominique J. P. Tilli, Ingeborg J. H. Vriens, Maaike de Boer, Vivianne C. G. Tjan-Heijnen

**Affiliations:** 1 Department of Medical Oncology, Maastricht University Medical Center, PO BOX 5800, 6202 AZ Maastricht, The Netherlands; 2 GROW-School for Oncology and Developmental Biology, Maastricht University, Maastricht, The Netherlands; 3 Department of Internal Medicine, Zuyderland Medical Center, Sittard-Geleen, The Netherlands; 4 Department of Medical Oncology, Amphia, Breda, The Netherlands; 5 Department of Medical Oncology, Jeroen Bosch Hospital, Den Bosch, The Netherlands; 6 Department of Internal Medicine, Catharina Hospital, Eindhoven, The Netherlands; 7 Department of Internal Medicine, Laurentius Hospital, Roermond, The Netherlands; 8 Department of Medical Oncology, Máxima Medical Center, Eindhoven, The Netherlands; 9 Department of Internal Medicine, Elkerliek Hospital, Helmond, The Netherlands; 10 Department of Internal Medicine, Sint Jans Gasthuis, Weert, The Netherlands; 11 Department of Internal Medicine, St Anna Hospital, Geldrop, The Netherlands

**Keywords:** Breast neoplasms, Survival, Metastatic breast cancer, HER2-positive disease, Pertuzumab, T-DM1

## Abstract

**Purpose:**

Immediate and proper implementation of a new and more potent therapy is important to ensure that the patient achieves the best possible outcome. This study aimed to examine whether the real-world overall survival (OS) has improved in patients with human epidermal growth factor receptor 2-positive (HER2 +) advanced breast cancer (ABC) since the market release of pertuzumab and T-DM1. Furthermore, we aimed to assess the implementation and survival rates per hormone receptor (HR) subtype.

**Patients and methods:**

We included 493 systemically treated patients consecutively diagnosed with HER2 + ABC in 2008–2017 from the SOutheast Netherlands Advanced BREast cancer (SONABRE) Registry. Median OS was obtained using the Kaplan–Meier method and differences between periods (2008–2012 versus 2013–2017) were tested using multivariable Cox proportional hazards regression modeling. The 3-year implementation rates were estimated for any HER2-targeted therapy, pertuzumab, and T-DM1 by using the competing risk method and calculated from the date of diagnosis of ABC to start of HER2-targeted therapy of interest.

**Results:**

The median OS in 2008–2012 versus 2013–2017 was 28.3 versus 39.7 months in all patients (adjusted hazard ratio (adjHR) 0.85, 95%CI 0.66–1.08), 29.9 versus 36.3 months in patients with HR + /HER2 + disease (adjHR 0.97, 95%CI 0.72–1.32), and 22.7 versus 40.9 months in patients with HR-/HER2 + disease (adjHR 0.59, 95%CI 0.38–0.92). Any HER2-targeted therapy was used in 79% of patients in 2008–2012 and in 84% in 2013–2017. The use of pertuzumab and T-DM1 in 2013–2017 was 48% and 29%, respectively. For patients diagnosed with HR + /HER2 + and HR-/HER2 + disease, implementation rates in 2013–2017 were , respectively, 77% and 99% for any HER2-targeted therapy, 38% and 69% for pertuzumab, and 24% and 40% for T-DM1.

**Conclusion:**

The survival of patients with HER2 + ABC improved since the introduction of pertuzumab and T-DM1. There is room for improvement in implementation of these HER2-targeted therapies, especially in patients with HR + /HER2 + disease.

**Supplementary Information:**

The online version contains supplementary material available at 10.1007/s10549-021-06178-8.

## Introduction

Approximately one-fifth of patients with advanced breast cancer (ABC) have human epidermal growth factor receptor 2-positive (HER2 +) disease [1, 2]. Before the introduction of HER2-targeted therapy, patients with HER2 + disease tended to have an aggressive disease course resulting in a poor prognosis [1]. However, after the introduction of trastuzumab in the year 2000, the overall survival (OS) of patients with HER2 + ABC substantially improved [3–10].

More recently, pertuzumab was approved by the Food and Drug Administration (FDA) in 2012, and the European Medicines Agency (EMA) in 2013, as first-line HER2-targeted therapy combined with trastuzumab plus taxane, after the CLEOPATRA trial had shown an impressive median OS gain of 16.3 months [11]. Shortly thereafter, the EMILIA trial assessed the efficacy of trastuzumab-emtansine (T-DM1) compared with lapatinib plus capecitabine in patients who were previously treated with trastuzumab and taxane [12]. T-DM1 prolonged median OS significantly by 5.8 months, after which it was implemented for patients with progressive disease on at least one palliative line of trastuzumab-based systemic therapy. Efficacy of T-DM1 was also confirmed in the TH3RESA trial in patients who had received at least two prior palliative HER2-targeted therapies, showing a median survival gain of 6.9 months compared with the treatment of physician’s choice [13]. T-DM1 showed comparable survival results as with taxane plus trastuzumab in the first line, whereas the addition of pertuzumab to T-DM1 did not increase the efficacy [14]. Hence, pertuzumab in combination with trastuzumab and taxane became the standard of care as first-line treatment and T-DM1 gained a position as a second and further line of treatment.

To our knowledge, only one real-world study, using the French ESME cohort, looked at survival trends in ABC including the period where pertuzumab and T-DM1 were introduced [9]. They observed steadily improving survival rates in patients diagnosed from 2008 through 2014. The investigators hypothesized that their findings might be related to the market release of new HER2-targeted drugs, although they did not formally test this. Indeed, the impact of pertuzumab and T-DM1 may have been small as during their study these drugs were only just introduced. Conversely, trastuzumab in the early disease setting was implemented very rapidly due to the impressive effectiveness of the drug [15, 16]. Implementation patterns of new HER2-targeted systemic therapies in ABC are not reported, so far.

The purpose of this study was therefore to examine whether OS has improved in relation to the market release of pertuzumab and T-DM1, by comparing a 5-year period before (2008–2012) and after (2013–2017) the introduction of these two drugs. Furthermore, we aimed to assess the survival rates and implementation of pertuzumab, and T-DM1 per hormone receptor (HR) subtype, as we have shown before that first-line systemic treatment choices for HER2 + disease differs by HR status [18].

## Patients and methods

### SOutheast Netherlands Advanced BREast cancer (SONABRE) registry

Data for this study were obtained from the SONABRE Registry (NCT-03577197). This is an ongoing observational cohort study, which aims to include all patients diagnosed with ABC de novo or during follow-up after early-stage breast cancer from hospitals in the Southeast of the Netherlands. Information, including patient and tumor characteristics, treatment information (surgery, radiotherapy, and systemic treatment, (neo-) adjuvant and palliative), response to systemic therapy, and date, and cause of death, is collected from medical files by specially trained registration clerks. The SONABRE Registry has already been effectively used to perform real-world studies on prognosis, effectiveness, and safety of treatment for ABC [2, 17–20]. The Medical Research Ethics Committee of Maastricht University Medical Centre approved the registry (15-4-239).

### Patients

We selected patients diagnosed with ABC in nine hospitals, comprising of one academic, five teachings, and three non-teaching hospitals. Patients were included when diagnosed with HER2 + ABC from January 2008 through December 2017 for eight hospitals, and one hospital from January 2010 through December 2017. Data lock was on February 14, 2020. Patients who received systemic therapy were eligible for analyses, whereas patients not receiving palliative systemic therapy were excluded. HER2 positivity was defined as a positive fluorescence in situ hybridization (FISH) result or an immunohistochemistry score of 3 + . HR (estrogen/progesterone receptor) positivity was defined as positive nuclear staining of ≥ 10% of one or both receptors. To determine the HR/HER2 status , we used information from the metastatic site (41%), and if not available, from a prior recurrence or primary tumor (59%).

### Endpoints and statistical analyses

Our main objective was to determine whether OS has improved since the introduction of pertuzumab and T-DM1 in 2013 by comparing patients diagnosed with HER2 + ABC 5 years before and after the introduction of these new drugs (2008–2012 versus 2013–2017). OS was defined as the time from date of diagnosis of ABC to date of death or censored at the date of last update. Survival analyses were performed using the Kaplan–Meier method, log-rank tests, and multivariable Cox proportional hazards regression modeling. The prognostic factors included were incidence period, age per year, performance status, HR status, number of initial metastatic sites, initial metastatic sites, and metastatic-free interval (MFI).

The secondary objective was to determine the rate of implementation of any HER2-targeted therapy, pertuzumab, and T-DM1 for the period of 2008–2012 and 2013–2017 and by year of diagnosis. Cumulative use at 3-year beyond ABC diagnosis of any HER2-targeted therapy, pertuzumab, and T-DM1 were assessed by using competing risk analyses. The use of HER2-targeted therapy of interest was defined as ‘event’ and death without HER2-targeted therapy of interest as ‘competing event.’ Patients in follow-up were censored at the date of the last update. The number at risk consisted of patients still alive who did not (yet) receive the HER2-targeted therapy of interest. As we expected that HR status might influence the implementation of pertuzumab and T-DM1 in the real world, we additionally looked at the implementation rate, treatment pattern for the first three lines of systemic therapy, and outcome per HR subtype [8, 21, 22].

The main reason for the non-use of specified HER2-targeted therapy was determined in systemically treated and deceased patients, excluding patients who had died before the market release of pertuzumab (July 30, 2013) and T-DM1 (November 15, 2013).

Baseline characteristics for patients diagnosed in 2008–2012 and 2013–2017 were compared using the chi-square test for categorical variables and Mann–Whitney *U*-test for continuous variables. All reported P-values are two-sided and considered statistically significant at a value of ≤ 0.05.

## Results

### Baseline characteristics per period

**Fig. 1:**
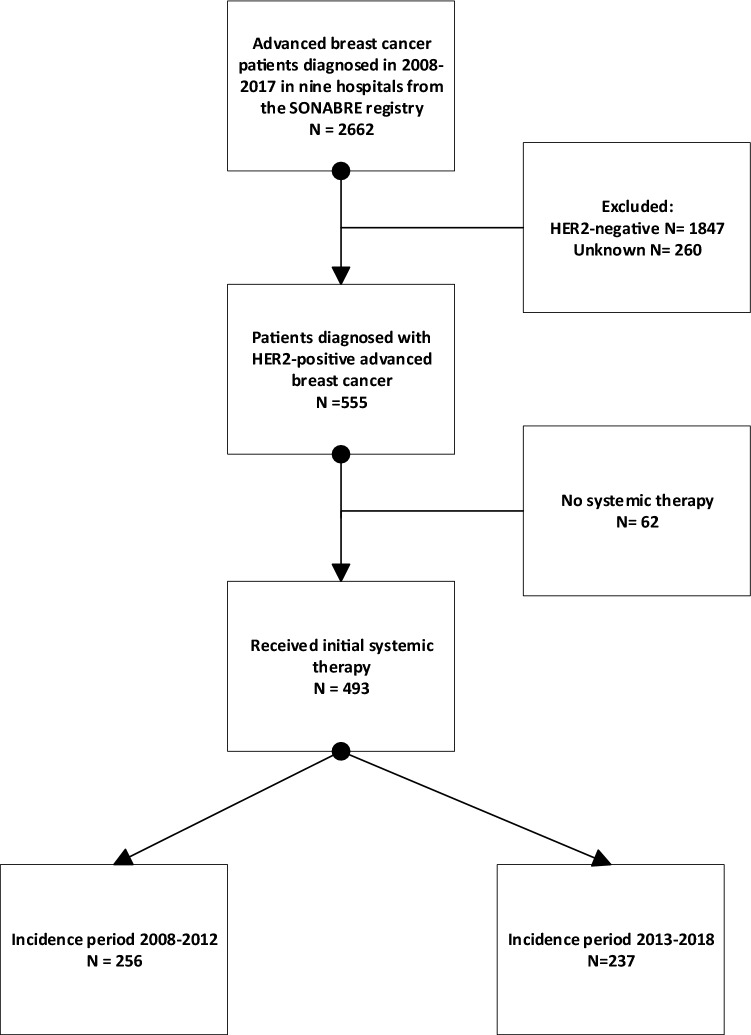
Flow chart study population

A total of 555 patients diagnosed with HER2 + ABC in 2008–2017 were identified, of whom 493 (89%) received systemic therapy (Fig. 1). Of these 493 eligible patients, 256 were diagnosed in 2008–2012 and 237 in 2013–2017. Of all patients, 67% were diagnosed with HR + /HER2 + disease and 33% with HR − /HER2 + disease (Table 1). The median follow-up duration was 74 months (95% confidence interval (CI) 63–85), during which 352 (71%) patients had died, and 6 (1%) patients were lost to follow-up due to transfer to a non-participating hospital.

**Table 1:** Baseline characteristics of systemically treated patients with HER2-positive advanced breast cancer (ABC) in 2008–2012 versus 2013–2017

	Period (year of ABC diagnosis)		*P*
	2008–2012 *N* = 256	2013–2017 *N* = 237	
Characteristics	*N* (%)	*N* (%)	
Age at diagnosis ABC			0.10
< 75 years	228 (89)	199 (84)	
≥ 75 years	28 (11)	38 (16)	
Median (95% CI)	60 (58–61)	58 (58–61)	0.67
Comorbidity^a^			
Any	115 (45)	102 (43)	0.67
Cardiovascular	83 (32)	56 (24)	0.03
Diabetes	30 (12)	22 (9)	0.38
Lung disease	23 (9)	18 (8)	0.58
Cerebrovascular	10 (4)	15 (6)	0.22
Non-breast malignancy	14 (6)	16 (7)	0.55
WHO performance score			0.30
WHO 0–1	124 (89)	177 (85)	
WHO ≥ 2	16 (11)	32 (15)	
Missing	116	28	
Hormone receptor status			0.52
HR +	168 (66)	162 (68)	
HR−	88 (34)	75 (32)	
Number of initial metastatic sites			0.91
Single organ	106 (41)	97 (41)	
Multiple organs	150 (59)	140 (59)	
Initial metastatic sites^a^			
Bone	171 (67)	148 (62)	0.31
Lymph node and soft tissue^b^	93 (36)	108 (46)	0.04**
Visceral^c^	167 (65)	146 (62)	0.40
CNS^d^	27 (11)	24 (10)	0.89
Metastatic-free interval			0.56
< 3 months/ de novo	75 (29)	80 (34)	
3–23 months	39 (15)	35 (15)	
≥ 24 months	142 (56)	122 (51)	
(Neo-)adjuvant therapy^a,e^			
Yes	150 (83)	130 (83)	0.99
HER2-targeted therapy	71 (39)	72 (46)	0.22
Pertuzumab-based therapy	1 (1)	3 (2)	0.25
Endocrine therapy	95 (53)	102 (65)	0.02
Chemotherapy	122 (67)	100 (64)	0.47
No	31 (17)	27 (17)	

*ABC* advanced breast cancer, *CNS* central nervous system, *HR* hormone receptor, *HER2* human epidermal growth factor receptor 2, *WHO* World Health Organization

**The observed statistically significant difference may be explained by the difference in definition of soft tissue in the period 2008–2012

^a^Sum of percentages exceeds 100 because multiple options are possible

^b^Lymph nodes, skin, and eye

^c^Liver, lung, pleura, peritoneum, gastrointestinal track, kidney, adrenal, and ovaries

^d^Brain and leptomeningeal

^e^Among patients with recurrent metastases (excluding patients with de novo ABC)

Table 1 shows the baseline characteristics categorized by the period of diagnosis of distant metastases. Overall, patients diagnosed in 2008–2012 tended to be slightly more often diagnosed with cardiovascular comorbidity (32% versus 24%, *P* = 0.03), and less often with lymph node and soft tissue metastasis (36% versus 46%, *P* = 0.04), and also less often treated with (neo-) adjuvant endocrine therapy (53% versus 65%, *P* = 0.02) when compared with the more recent period in 2013–2017. When comparing diagnosis periods with regard to HR status, patients with HR + /HER2 + disease tended to be older in the more recent period (≥ 75 years, 11% versus 19%, *P* = 0.05), whereas patients with HR-/HER2 + disease were less frequently diagnosed with visceral metastasis (77% versus 59%, *P* = 0.01) and more often with soft tissue metastasis (43% versus 59%, *P* = 0.05) in the more recent period (Supplemental Table S1).

### Overall survival

**Fig. 2:**
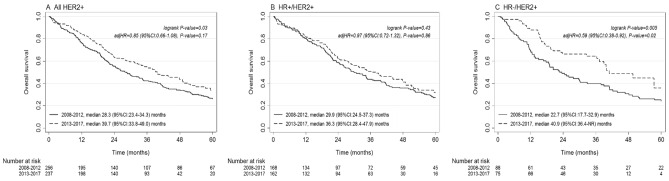
Overall survival in patients systemically treated for **a** HER2 + , **b** HR + /HER2 + , **c** HR-/HER2 + ABC by incidence period of ABC diagnosis

The median OS of patients diagnosed in 2008–2012 with HER2 + ABC was 28.3 months (95% CI 23.4–34.3), as compared with 39.7 months (95% CI 33.8–49.0) for those diagnosed in 2013–2017 (log-rank *P* = 0.03), a difference of 11.4 months (Fig. 2a). Among patients with HR + /HER2 + disease, the median OS was not statistically significant different, 29.9 months (95% CI 24.5–37.3) for those diagnosed in 2008–2012 and 36.3 months (95% CI 28.4–47.9) for those diagnosed in 2013–2018 (log-rank *P* = 0.43) (Fig. 2b). Contrarily, in patients with HR − /HER2 + disease, the median OS was 22.7 months (95% CI 17.7–32.9) when diagnosed in 2008–2012 and 40.9 months (95% CI 36.4-not reached) when diagnosed in 2013–2017 (log-rank *P* = 0.005), an improvement in median OS of 18.2 months (Fig. 2c).

In the multivariable model including patients with HER2 + ABC, the following variables had a significant (≤ 0.05) or borderline significant (≤ 0.10) impact on OS: age at ABC diagnosis (hazard ratio per year 1.01, 95% CI 1.01–1.02), WHO performance status ≥ 2 (hazard ratio 2.29, 95% CI 1.58–3.32), WHO performance status unknown (hazard ratio 1.61, 95% CI 1.27–2.05), multiple initial metastatic sites (hazard ratio 1.68, 95% CI 1.25–2.25), and MFI 3–23 months (hazard ratio 1.56, 95% CI 1.12–2.19) (Table 2). The incidence period 2013–2017 was no longer significantly associated with a longer survival time in patients with HER2 + total disease (hazard ratio 0.85, 95% CI 0.66–1.08). Among patients with HR-/HER2 + disease, incidence period 2013–2017 continued to be associated with a significant longer OS when compared with 2008–2012 (hazard ratio 0.59, 95% CI 0.38–0.92).

**Table 2:** Multivariable analysis for overall survival (OS) in patients who received at least one line of palliative systemic therapy

	HER2 + total *N* = 493, events = 352			HR + /HER2 + *N* = 330, events = 242			HER2 + total *N* = 493, events = 352		
	Hazard ratio	95% CI	*P*	Hazard ratio	95% CI	*P*	Hazard ratio	95% CI	*P*
Incidence period									
2008–2012	Ref			Ref			Ref		
2013–2017	0.85	0.66–1.08	0.17	0.87	0.72–1.32	0.86	0.59	0.38–0.92	0.02
Age at diagnosis ABC									
Age per year	1.01	1.01–1.02	0.002	1.02	1.01–1.03	0.001	1.00	0.98–1.02	0.97
WHO performance status									
0–1	Ref			Ref			Ref		
≥ 2	2.29	1.58–3.32	< 0.001	2.89	1.83–4.57	< 0.001	1.34	0.68–2.64	0.40
Unknown	1.61	1.27–2.05	< 0.001	1.74	1.30–2.33	< 0.001	1.48	0.95–2.30	0.08
Hormone receptor									
Positive	Ref			NA			NA		
Negative	0.86	0.68–1.08	0.19	NA			NA		
Number initial metastatic sites									
Single organ	Ref			Ref			Ref		
Multiple organs	1.68	1.25–2.25	0.001	1.46	1.02–2.09	0.04	2.14	1.30–3.53	0.003
Initial metastatic sites									
Bone only	Ref			Ref			Ref		
Soft tissue without visceral or CNS^a^	0.80	0.49–1.31	0.38	0.94	0.53–1.65	0.82	0.71	0.25–2.02	0.52
Visceral without CNS^b^	1.23	0.85–1.78	0.26	1.31	0.86–2.00	0.21	1.27	0.55–2.92	0.58
CNS^c^	1.36	0.84–2.20	0.21	1.94	1.10–3.42	0.02	1.06	0.38–2.92	0.91
Metastatic-free interval									
< 3 months/ de novo	Ref			Ref			Ref		
3–23 months	1.56	1.12–2.19	0.009	1.84	1.20–2.84	0.005	1.46	0.79–2.70	0.23
≥ 24 months	1.07	0.83–1.37	0.61	1.14	0.84–1.54	0.39	0.91	0.56–1.47	0.69

*ABC* advanced breast cancer, *CI* confidence interval, *CNS* central nervous system, *HR* hormone receptor, *HER2* human epidermal growth factor receptor 2, *NA* not applicable

^a^Lymph nodes, skin and eye

^b^Liver, lung, pleura, peritoneum, gastrointestinal track, kidney, and ovaries

^c^Brain and leptomeningeal

Supplementary Figure S1 shows the OS per year, categorized by hormone receptor status. The log-rank *P*-value for the trend in OS per year was significant in all patients with HER2 + and in patients with HR − /HER2 + disease (*P* = 0.001 and *P* = 0.003, respectively) and borderline significant in patients with HR + /HER2 + disease (*P* = 0.08). The 3-year OS rate in all patients with HER2 + disease was 34% (95% CI 22–46%) when diagnosed with ABC in 2008 and 65% (95% CI 46–78%) when diagnosed in 2017. Patients with HR + /HER2 + and HR − /HER2 + disease had a 3-year OS rate of , respectively, 34% (95% CI 18–46%) and 33% (95% CI 15–53%) when diagnosed in 2008, and 56% (95% CI 12–31%) and 77% (95% CI 53–90%) when diagnosed in 2017.

### Implementation of HER2-targeted therapy

**Fig. 3:**
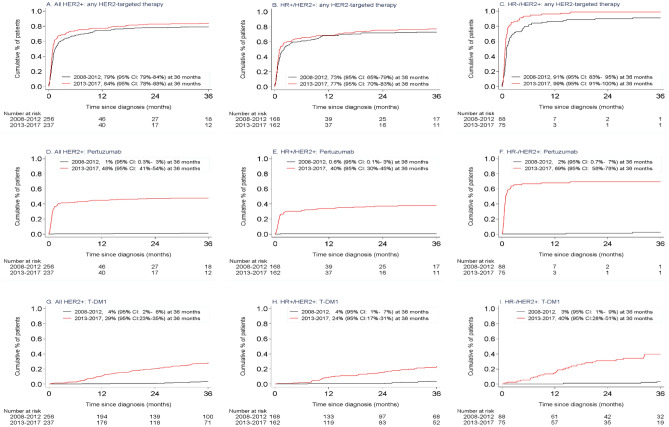
Use of **a**–**c** any HER2-targeted therapy, **d**–**f** pertuzumab-based therapy, and **g**–**i** T-DM1 in systemically treated patients and categorized by incidence period and hormone receptor status of

Figure 3 and Supplementary Table S2 show the implementation rates for the incidence periods 2008–2012 and 2013–2017, and Supplemental Figure S2 shows the implementation per year. Of systemically treated patients with HER2 + disease, cumulative 3-year use of any HER2-targeted therapy was 79% (95% CI 78–88%) when diagnosed in 2008–2012 and 84% (95% CI 78–88%) when diagnosed in 2013–2017, 3-year use of pertuzumab was 1% (95% CI 0.3–3%) and 48% (95% CI 41–54%), and 3-year use of T-DM1 was 4% (95% CI 2–6%) and 29% (95% CI 23–35%), respectively. For patients with HR + /HER2 + disease, the implementation rates in 2008–2012 and 2013–2017 were 73% (95% CI 65–79%) versus 77% (95% CI 70–83%) for any HER2-targeted therapy, 0.6% (95% CI 0.1–3%) versus 38% (95% CI 30–45%) for pertuzumab-based therapy, and 4% (95% CI 1–7%) versus 24% (95% CI 17–31%) for T-DM1. For patients with HR-/HER2 + disease, the rates in 2008–2012 and 2013–2017 were 91% (95% CI 83–95%) versus 99% (95% CI 91–100%) for any HER2-targeted therapy, 2% (95% CI 0.7–7%) versus 69% (95% CI 58–78%) for pertuzumab-based therapy, and 3% (95% CI 1–9%) versus 40% (95% CI 28–51%) for T-DM1, respectively. Among 29 patients who were not treated with HER2-targeted therapy before death, 52% had a contra-indication for chemo- and/or HER2-targeted therapy, and for the other 48%, no reason was specified. The reason for non-use was not documented for 70% of the 75 patients who had not received pertuzumab, and for 76% of the 17 patients who had not received T-DM1, whereas approximately two-thirds of these had received trastuzumab during their advanced disease course.

Table 3 shows the treatment pattern for the first three lines of systemic therapy categorized by HR status and incidence period. Among patients with HR + /HER2 + disease, the use of HER2-targeted therapy remained similar in lines one through three between the incidence period 2008–2012 and 2013–2017. Around one-third of patients with HR + /HER2 + disease were treated with endocrine monotherapy across all three lines of therapy, irrespective of incidence period. In patients with HR-/HER2 + disease, the use of HER2-targeted therapy increased from approximately 80% in 2008–2012 to 90% in 2013–2017.

**Table 3:** Treatment pattern, categorized by HR status and incidence period

HER2 + total	*N*	HER2-targeted	ET	CT
		%	%	%
1st line				
2008–2012	256	62	24	14
2013–2017	237	69	27	4
2nd line				
2008–2012	189	70	21	9
2013–2017	164	70	23	7
3rd line				
2008–2012	134	67	19	15
2013–2017	99	73	21	6
1st line				
2008–2012	168	53	36	11
2013–2017	162	58	38	4
2nd line				
2008–2012	134	66	30	4
2013–2017	123	64	29	7
3rd line				
2008–2012	102	61	24	15
2013–2017	80	69	25	6

HR-/HER2 +	*N*	HER2-targeted	ET^a^	CT
		%	%	%
1st line				
2008–2012	88	80	1	19
2013–2017	75	93	3	4
2nd line				
2008–2012	55	80	0	20
2013–2017	41	90	5	5
3rd line				
2008–2012	32	81	0	19
2013–2017	19	90	5	5

*CT* chemotherapy, *ET* endocrine therapy, *HR* hormone receptor, *HER2* human epidermal growth factor receptor

^a^Includes 3 patients with HR + primary breast tumor

## Discussion

The value of real-world studies lies in providing insight into the use and effects of new drugs without strict eligibility criteria, thereby helping physicians to interpret and generalize existing data when making treatment decisions [23, 24]. In this ongoing real-world study from the SONABRE registry, we identified 493 systemically treated patients consecutively diagnosed with HER2 + ABC in the period 2008–2017. Here, we present the trend in survival and implementation by comparing the 5-year diagnosis period before (2008–2012) and after (2013–2017) the introduction of pertuzumab and T-DM1. The median OS improved significantly by 11 months in all patients with HER2 + disease. The improvement in OS was particularly evident in the HR-/HER2 + group (median gain of 18 months), while in patients with HR + /HER2 + disease survival remained rather similar. We observed this survival improvement even though the implementation of pertuzumab and T-DM1 was lower than anticipated. Overall, the use of pertuzumab and T-DM1 among patients diagnosed with ABC in 2013–2017 was 48% and 29%, respectively. Implementation of pertuzumab started in the incidence year 2013 at 19%, increased to 41% in 2014 and 56% in 2015 and was constant afterward (61–64%). The use of T-DM1 was prominent as of 2013 (26–33%). One may hypothesize that non-users may have missed out on the survival benefits of pertuzumab or T-DM1.

We observed a median OS of 40 months for patients systemically treated for HER2 + ABC in 2013–2017, which was shorter than the French ESME cohort reporting a median OS of 50 months for the period 2008–2016 [10]. This discrepancy in OS may be caused by differences in screening for ABC and patient selection. Half of the metastases in the ESME population were found asymptomatically (through screening), whereas screening for distant metastases is not standard practice in the Netherlands. This advancement in diagnosis may explain part of the longer OS observed in the ESME population. In addition, ESME covers 18 Comprehensive Cancer Centers (CCCs), whereas a variety of hospital types are participating in SONABRE. We were not able to analyse the influence of hospital type on survival because the number of patients and hospitals (only one academic hospital) were too small. Nevertheless, one cannot rule out the possibility that the lower use of HER2-targeted therapy plus chemotherapy in the Netherlands also contributes to the lower OS observed in the SONABRE population.

Notably, the survival gain observed in our cohort resembles the results of prior randomized controlled trials [11, 12]. In the CLEOPATRA trial, the addition of pertuzumab to trastuzumab and docetaxel led to an increase in median OS of 16 months compared with 11 months in our SONABRE cohort [11]. In the EMILIA trial, where T-DM1 was compared with capecitabine/lapatinib, prior use of pertuzumab was not permitted and randomization occurred during the disease course making this study difficult to compare with our results [12]. Nevertheless, it is worthy to note that the implementation rate increased each subsequent incidence year and for those diagnosed in 2017, we found a 3-year OS rate of 65% (95% CI 46–78%), equivalent to the 3-year OS rate of 65.8% (95%CI 59.8–71.7%) observed in the pertuzumab arm of CLEOPATRA. The similarity in outcome is quite surprising. Study patients are generally highly selected, consisting of on average 5% of the real-world patient population [25]. Indeed, inclusion criteria in the CLEOPATRA consisted of performance score ≤ 1, no CNS, no uncontrolled medical condition, and an interval of at least 12 months between completion of the (neo-) adjuvant therapy and the diagnosis of ABC, resulting in patients with more favorable baseline characteristics when compared with our real-world population. A similar 3-year survival rate is even more intriguing when considering the fact that pertuzumab was implemented in only 64% of the patients diagnosed with ABC in 2017 (Supplemental Figure S2). It remains, however, uncertain how much higher the OS rate would have been with better implementation.

Interestingly, implementation rates and OS differed with regard to HR status. For patients diagnosed with HR + /HER2 + and HR-/HER2 + disease, implementation rates in 2013–2017 were , respectively, 38% and 69% for pertuzumab and 24% and 40% for T-DM1. Even though the implementation rates for pertuzumab and T-DM1 were almost twice as high for patients with HR-/HER2 + disease when compared with HR + /HER2 + disease, they were overall lower than expected. In real life, the implementation rates will never reach 100% due to the contra-indication for HER2-targeted therapy plus chemotherapy, such as comorbidity (renal dysfunction, cardiovascular disease), performance status > 2, and patient preferences. Nevertheless, there is still room for improvement, especially in HR + /HER2 + disease where the use of HER2-targeted therapy has hardly increased over the years. In the Netherlands, pertuzumab and T-DM1 are fully reimbursed to facilitate the use of these new and expensive agents. However, we are quite conservative in using chemotherapy-based therapy as first-line treatment and prefer endocrine-based therapy for patients with HR + disease and mild symptoms. This could also explain the lower implementation of pertuzumab and T-DM1 found in HR + than in HR-/HER2 ABC and could have led to a missed opportunity to benefit from these new drugs.

The strength of our prospective cohort study lies in the unselected inclusion of all systemically treated patients diagnosed with HER2 + ABC from a ten-year inclusion period in nine different hospitals. The data were manually screened and collected by specially trained registration clerks, which contributed to the high quality of the data. Another strength that sets us apart from other registry studies [8, 9, 26, 27] is that our cohort consists of more recently diagnosed patients with HER2 + ABC, showing a more current treatment pattern, with a substantial long median follow-up duration of 74 months. Our study also has some limitations, inherent to the observational character of the study and by using medical files for the data collection, reasons for non-use are not always clearly documented. A survey study should be conducted on how treatment decisions are made. In addition, the number of patients diagnosed with HR + /HER2 + disease is limited (*N* = 330) to observe small OS differences. Nonetheless, this study is an important addition to the evolving demand for real-world studies showing implementation and treatment patterns of new targeted therapies [23, 28]. The reported implementation rate and patient’s OS presented in this real-world study were unbiased.

The outcomes presented in this study, providing a realistic implementation pattern, can be utilized for multiple settings, such as budget impact analysis and market penetration estimates of new drugs. Our results also show that more research needs to be done on how treatment decisions are made in daily practice and their subsequent effects on treatment patterns and outcomes. Additionally, physicians need more guidance on how to decide which patients may or may not benefit from new therapies. Previously, a real-world study in stage IV colorectal cancer patients showed that the OS of non-eligible non-trial patients had a significantly worse outcome (HR 1.70, *P* < 0.01) when compared with trial patients [29]. These results emphasize the need for external validation of trial outcomes. Furthermore, we presented clear differences in survival and implementation rates with regard to HR status. The difference by HR status suggests either a biological impact, an impact of chosen systemic therapies, or differential effectiveness of systemic therapies used. Real-world data on treatment choices and outcomes from an unselected patient population are important to gain more clinical insight into this complex interplay.

## Conclusion

Survival of patients with HER2 + ABC improved after the introduction of pertuzumab and T-DM1. The slow and limited implementation of pertuzumab and T-DM1 could have led to a missed survival benefit in some patients. Future studies elucidating the indicators for making systemic treatment choices, especially in HR + /HER2 + disease, may provide insights into current medical decision-making and assist physicians in making individual treatment choices.

## Supplementary Information

Below is the link to the electronic supplementary material.

Supplementary file1 (PPTX 230 kb)

Supplementary file2 (PPTX 405 kb)

Supplementary file3 (DOCX 16 kb)

Supplementary file4 (DOCX 13 kb)

Supplementary file5 (DOCX 12 kb)

## Data Availability

Data are available upon request (vcg.tjan.heijnen@mumc.nl).
