# Supplementary figures and images for: Survival before and after the introduction of pertuzumab and T-DM1 in HER2-positive advanced breast cancer, a study of the SONABRE Registry
Source: Breast Cancer Res Treat. 2021 Mar 20;188(2):571–81. doi: 10.1007/s10549-021-06178-8 (PMC8260428; doi:10.1007/s10549-021-06178-8)

## Slide 1
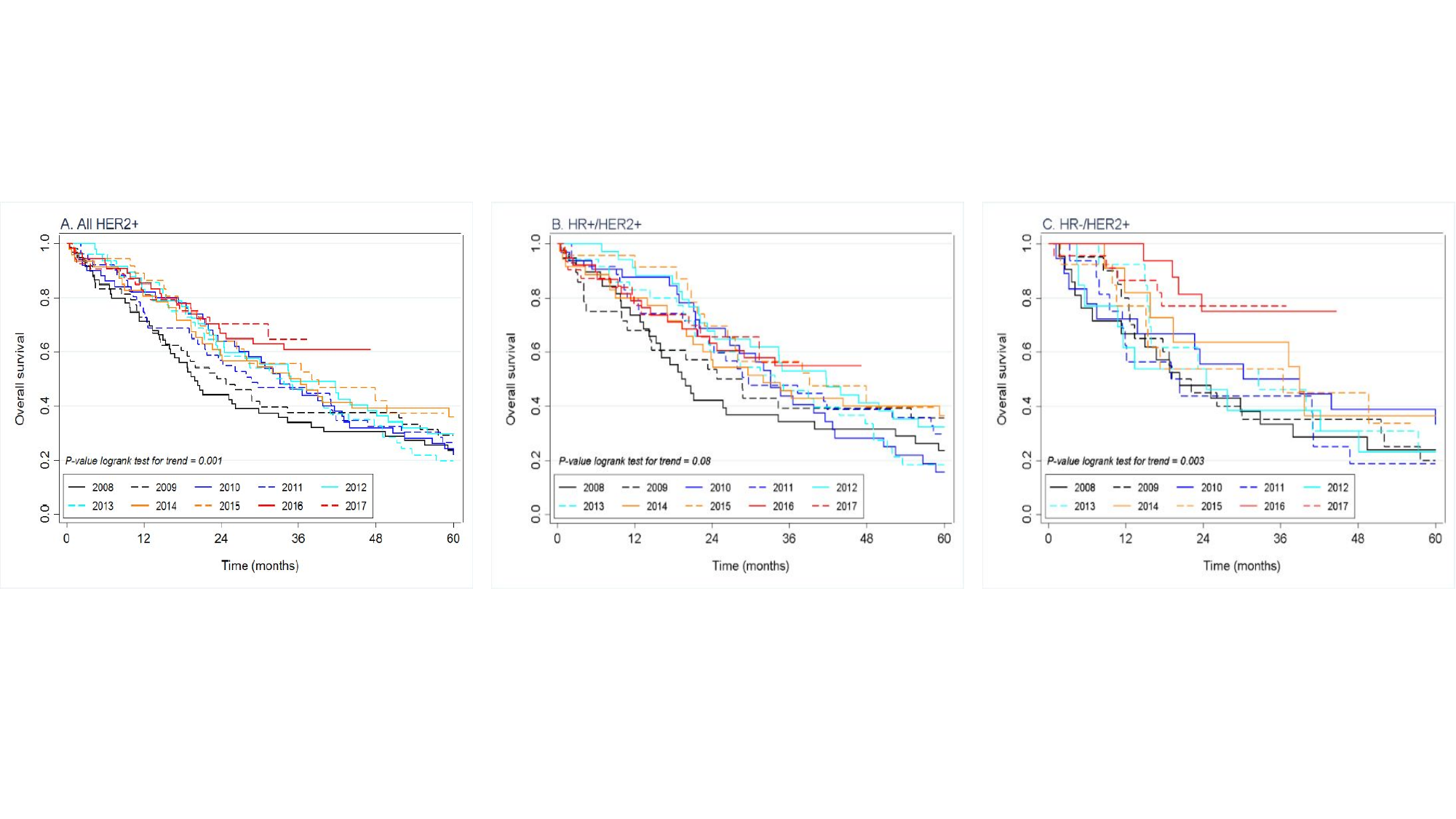

Supplement: Supplementary file 1 — Supplementary file1 (PPTX 230 kb) [file 10549_2021_6178_MOESM1_ESM.pptx]

## Slide 1
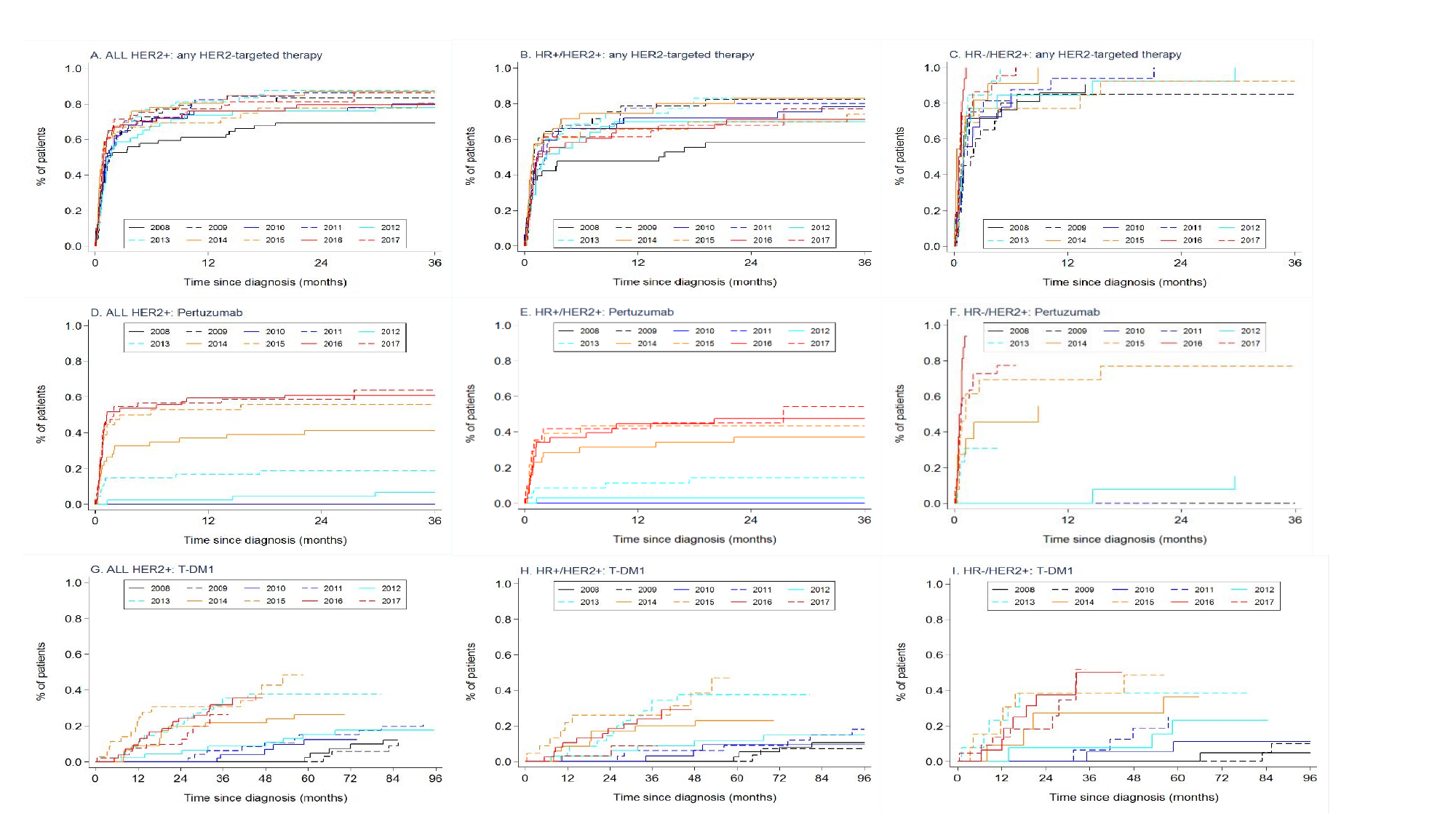

Supplement: Supplementary file 2 — Supplementary file2 (PPTX 405 kb) [file 10549_2021_6178_MOESM2_ESM.pptx]
