# Supplementary material for: Survival before and after the introduction of pertuzumab and T-DM1 in HER2-positive advanced breast cancer, a study of the SONABRE Registry
Source: Breast Cancer Res Treat. 2021 Mar 20;188(2):571–81. doi: 10.1007/s10549-021-06178-8 (PMC8260428; doi:10.1007/s10549-021-06178-8)
Supplement: Supplementary file 3 — Supplementary file3 (DOCX 16 kb) [file 10549_2021_6178_MOESM3_ESM.docx]

**Supplementary Table S1.** Baseline characteristics of systemically treated patients with HER2-positive advanced breast cancer (ABC) diagnosed in 2008-2017, categorized by hormone receptor status

|  |  | **HR+/HER2+** | | | | **HR-/HER2+** | | | |
| --- | --- | --- | --- | --- | --- | --- | --- | --- | --- |
|  |  | *2008-2012*  *N=168* |  | *2013-2017*  *N=162* | *P* | *2008-2012*  *N=88* |  | *2013-2017*  *N=75* | *P* |
| **Characteristics** | | *N (%)* |  | *N (%)* |  | *N (%)* |  | *N (%)* |  |
| **Age at diagnosis ABC** | |  |  |  | 0.05 |  |  |  | 0.85 |
|  | <75 years | 149 (89) |  | 131 (81) |  | 79 (90) |  | 68 (91) |  |
|  | ≥75 years | 19 (11) |  | 31 (19) |  | 9 (10) |  | 7 (9) |  |
|  | Median (95% CI) | 60 (58-62) |  | 60 (58-63) | 0.96 | 58 (55-61) |  | 57 (55-60) | 0.85 |
| **Comorbidity^*^** | |  |  |  |  |  |  |  |  |
|  | Any | 74 (44) |  | 75 (46) | 0.68 | 41 (47) |  | 27 (36) | 0.17 |
|  | Cardiovascular | 54 (32) |  | 41 (25) | 0.17 | 29 (33) |  | 15 (20) | 0.06 |
|  | Diabetes | 20 (12) |  | 17 (11) | 0.69 | 10 (11) |  | 5 (7) | 0.30 |
|  | Lung disease | 13 (8) |  | 15 (9) | 0.62 | 10 (11) |  | 3 (4) | 0.08 |
|  | Cerebrovascular | 7 (4) |  | 13 (8) | 0.14 | 3 (3) |  | 2 (3) | 0.78 |
|  | Non-breast malignancy | 9 (5) |  | 14 (9) | 0.24 | 5 (6) |  | 2 (3) | 0.34 |
| **WHO performance score^*^** | |  |  |  | 0.10 |  |  |  | 0.61 |
|  | WHO 0-1 | 80 (91) |  | 119 (83) |  | 44 (85) |  | 58 (88) |  |
|  | WHO ≥2 | 8 (9) |  | 24 (17) |  | 8 (15) |  | 8 (12) |  |
|  | Missing | 80 |  | 19 |  | 36 |  | 9 |  |
| **Number initial metastatic sites** | |  |  |  | 0.89 |  |  |  | 0.92 |
|  | Single organ | 76 (45) |  | 72 (44) |  | 30 (34) |  | 25 (33) |  |
|  | Multiple organs | 92 (55) |  | 90 (56) |  | 58 (66) |  | 50 (67) |  |
| **Initial metastatic sites^*^** | |  |  |  |  |  |  |  |  |
|  | Bone | 127 (76) |  | 107 (66) | 0.06 | 44 (50) |  | 41 (55) | 0.55 |
|  | Soft tissue^1^ | 55 (33) |  | 64 (40) | 0.20 | 38 (43) |  | 44 (59) | 0.05 |
|  | Visceral^2^ | 99 (59) |  | 102 (63) | 0.45 | 68 (77) |  | 44 (59) | 0.01 |
|  | CNS^3^ | 16 (10) |  | 11 (7) | 0.37 | 11 (13) |  | 13 (17) | 0.39 |
| **Metastatic-free interval** | |  |  |  | 0.43 |  |  |  | 0.23 |
|  | <3 months/ *de novo* | 51 (30) |  | 56 (35) |  | 24 (27) |  | 24 (32) |  |
|  | 3-23 months | 25 (15) |  | 17 (10) |  | 14 (16) |  | 18 (24) |  |
|  | ≥ 24 months | 92 (55) |  | 89 (55) |  | 50 (57) |  | 33 (44) |  |
| **(Neo-)adjuvant therapy**§**^*^** | |  |  |  |  |  |  |  |  |
|  | Yes | 98 (84) |  | 91 (86) | 0.67 | 52 (81) |  | 39 (77) | 0.53 |
|  | HER2-targeted therapy | 37 (32) |  | 44 (42) | 0.13 | 34 (53) |  | 28 (55) | 0.85 |
|  | Pertuzumab-based therapy | 0 (0) |  | 1 (1) | 0.29 | 1 (2) |  | 2 (4) | 0.43 |
|  | Endocrine therapy | 82 (70) |  | 87 (82) | 0.04 | 13 (20) |  | 15 (29) | 0.26 |
|  | Chemotherapy | 73 (62) |  | 63 (59) | 0.65 | 49 (77) |  | 51 (73) | 0.62 |
|  | No | 19 (16) |  | 15 (14) |  | 12 (19) |  | 12 (23) |  |

ABC=advanced breast cancer, CNS=central nervous system, HR=hormone receptor, HER2=Human Epidermal growth factor Receptor 2, WHO=World Health Organization

^*^ Sum of percentages exceeds 100 because multiple options are possible

^1^ Lymph nodes, skin and eye

^2^ Liver, lung, pleura, peritoneum, gastrointestinal track, kidney and ovaries

^3^ Brain and leptomeningeal

§ Among patients with recurrent metastases (excluding patients with *de novo* ABC)
