# Supplementary material for: Survival before and after the introduction of pertuzumab and T-DM1 in HER2-positive advanced breast cancer, a study of the SONABRE Registry
Source: Breast Cancer Res Treat. 2021 Mar 20;188(2):571–81. doi: 10.1007/s10549-021-06178-8 (PMC8260428; doi:10.1007/s10549-021-06178-8)
Supplement: Supplementary file 4 — Supplementary file4 (DOCX 13 kb) [file 10549_2021_6178_MOESM4_ESM.docx]

**Supplementary Table S2**. Cumulative use of any HER2-targeted therapy, pertuzumab-based therapy and T-DM1 in patients with HER2+, HR+/HER2+ and HR-/HER2+ ABC, categorized by incidence period

|  | Time since diagnosis | **Any HER2-targeted therapy** | | **Pertuzumab-based therapy** | | **T-DM1** | |
| --- | --- | --- | --- | --- | --- | --- | --- |
|  |  | 2008-2012 | 2013-2017 | 2008-2012 | 2013-2017 | 2008-2012 | 2013-2017 |
|  | *Months* | *% (95% CI)* | *% (95% CI)* | *% (95% CI)* | *% (95% CI)* | *% (95% CI)* | *% (95% CI)* |
| **HER2+** | 3 | 61 (55-67) | 69 (62-74) | 1 (0-3) | 41 (35-48) | 0 (0-0) | 1 (0-3) |
|  | 12 | 74 (69-79) | 77 (71-82) | 1 (0-3) | 45 (38-51) | 0 (0-1) | 11 (7-15) |
|  | 24 | 78 (72-83) | 83 (77-87) | 1 (0-3) | 48 (41-53) | 1 (0-3) | 20 (16-26) |
|  | 36 | 79 (74-84) | 84 (78-88) | 1 (0-3) | 48 (41-54) | 4 (2-6) | 29 (23-35) |
|  | 60 | * | * | * | * | 8 (5-12) | 36 (29-43) |
| **HR+** | 3 | 56 (48-63) | 60 (53-68) | 1 (0-3) | 30 (23-37) | 0 (0-0) | 1 (0-3) |
|  | 12 | 68 (60-75) | 69 (61-75) | 1 (0-3) | 34 (27-41) | 1 (0-3) | 9 (5-14) |
|  | 24 | 72 (64-78) | 75 (68-81) | 1 (0-3) | 37 (30-45) | 1 (0-4) | 16 (11-22) |
|  | 36 | 73 (65-79) | 77 (70-83) | 1 (0-3) | 38 (30-45) | 4 (1-7) | 24 (17-31) |
|  | 60 | * | * | * | * | 7 (4-12) | 31 (23-40) |
| **HR-** | 3 | 73 (62-81) | 87 (77-93) | 1 (0-6) | 65 (53-74) | 0 (0-0) | 3 (1-8) |
|  | 12 | 86 (77-92) | 96 (88-99) | 1 (0-6) | 68 (56-77) | 0 (0-0) | 15 (8-24) |
|  | 24 | 90 (81-95) | 99 (91-100) | 1 (0-6) | 69 (57-78) | 1 (0-6) | 31 (21-42) |
|  | 36 | 91 (83-95) | 99 (91-100) | 2 (0-7) | 69 (57-78) | 3 (1-9) | 40 (28-51) |
|  | 60 | * | * | * | * | 10 (5-18) | 47 (33-60) |

CI=confidence interval, HR=hormone receptor, HER2=Human Epidermal growth factor Receptor 2

*The remaining number of patients at risk were too low for estimating cumulative use of HER2-targeted therapy of interest, because at that time patients either already received the therapy of interest or died without receiving it.
