# Supplementary material for: Survival before and after the introduction of pertuzumab and T-DM1 in HER2-positive advanced breast cancer, a study of the SONABRE Registry
Source: Breast Cancer Res Treat. 2021 Mar 20;188(2):571–81. doi: 10.1007/s10549-021-06178-8 (PMC8260428; doi:10.1007/s10549-021-06178-8)
Supplement: Supplementary file 5 — Supplementary file5 (DOCX 12 kb) [file 10549_2021_6178_MOESM5_ESM.docx]

**Supplementary Table S3.** Registration indication and date of approval for pertuzumab and T-DM1

|  | **FDA^1^** | **EMA^2^** | **NZa^3^** |
| --- | --- | --- | --- |
| **Agent** |  | | |
| **Pertuzumab** | July 9, 2012 | March 4, 2013 | July 30, 2013 |
|  | ^*^In combination therapy with trastuzumab and docetaxel, for the treatment of HER2+ locally advanced or metastatic breast cancer who have not previously received HER2-targeted therapy or chemotherapy for their metastatic disease. | | |
| **Trastuzumab-emtansine (T-DM1)** | February 22, 2013 | November 15, 2013 | June 26, 2014 |
|  | ^*^HER2 positive, non-resectable, locally advanced or metastatic breast cancer previously treated with trastuzumab and a taxane, individually or in combination. | | |

^1^ U.S. Food and Drug Administration

^2^ European Medicine Agency

^3^ Dutch Healthcare Authority

^*^Registration text EMA
